# The Use of Floseal Hemostatic Agent for Local Bleeding Control in Fungating Breast Cancer: A Case Report and Review of Literature

**DOI:** 10.7759/cureus.72962

**Published:** 2024-11-04

**Authors:** Hashim Abid, Mahmoud Soliman, Kathryn Williams

**Affiliations:** 1 Breast Surgery, North Manchester General Hospital, Manchester University NHS Foundation Trust, Manchester, GBR

**Keywords:** bleeding tumor, breast cancer, floseal, fungating breast mass, fungating tumor, hemostatic agent

## Abstract

Advanced breast cancer can present with bleeding tumors that are difficult to control using traditional techniques. The use of topical agents has been reported in the local management of bleeding malignant tumors. We report a challenging case of persistent bleeding in a 40-year-old female patient with metastatic breast cancer, presenting with significant uncontrollable bleeding from the fungating breast tumor where conventional measures failed to halt blood loss. We report the successful hemostasis using Floseal (topical hemostatic agent) in combination with local dressing. We recommend that topical application of Floseal should be considered in the management of uncontrolled bleeding due to fungating malignant wounds. Liaising with tissue viability specialists is essential for optimum management and other symptom control. Larger scale comparative studies are needed to further explore these findings.

## Introduction

The management of fungating and bleeding tumors represents one of the most complex challenges in the palliative care of cancer patients. Fungating tumors, also called ulcerating cancers and malignant wounds, develop when malignant cells from a primary or secondary cancer infiltrate the epithelium, creating a wound that is fungus-like in appearance. Breast cancer was reported to have the highest prevalence of these malignant wounds among all cancers [1]. The use of topical agents is described in the symptomatic management of pain, odor, discharge, and bleeding, and often requires off-label use of medication and dressings [2]. Herein, we report a case of metastatic breast cancer presenting with a fungating breast wound complicated with uncontrollable bleeding that was successfully managed using Floseal topical hemostatic agent.

## Case presentation

A 40-year-old woman presented to the emergency department of a district general hospital with a bleeding fungating wound locally infiltrating the whole of the right breast. She had no history of medical problems with no regular medications. On examination, there was a large fungating wound with necrotic tissue, malodorous exudate, and diffuse significant surface bleeding as well as palpable right axillary lymph nodes. Examination of the left breast and left axilla was unremarkable.

A punch biopsy from the wound confirmed a hormone receptor-negative, HER2 positive, grade 3 invasive ductal carcinoma with high proliferative index (Ki67, 70%). A staging CT scan of the chest, abdomen, and pelvis demonstrated the fungating right breast mass with necrotic ipsilateral axillary lymph nodes. Extensive pulmonary metastasis with metastatic mediastinal and hilar lymphadenopathy was confirmed.

Following a fast-track discussion in the local multidisciplinary team (MDT) meeting, the tumor was deemed inoperable, and the plan was to offer urgent referral to oncology services for palliative systemic therapy and consideration of urgent radiotherapy. Palliative care support was also provided through appropriate wound management, tissue viability specialist advice, and pain control.

**Figure 1:**
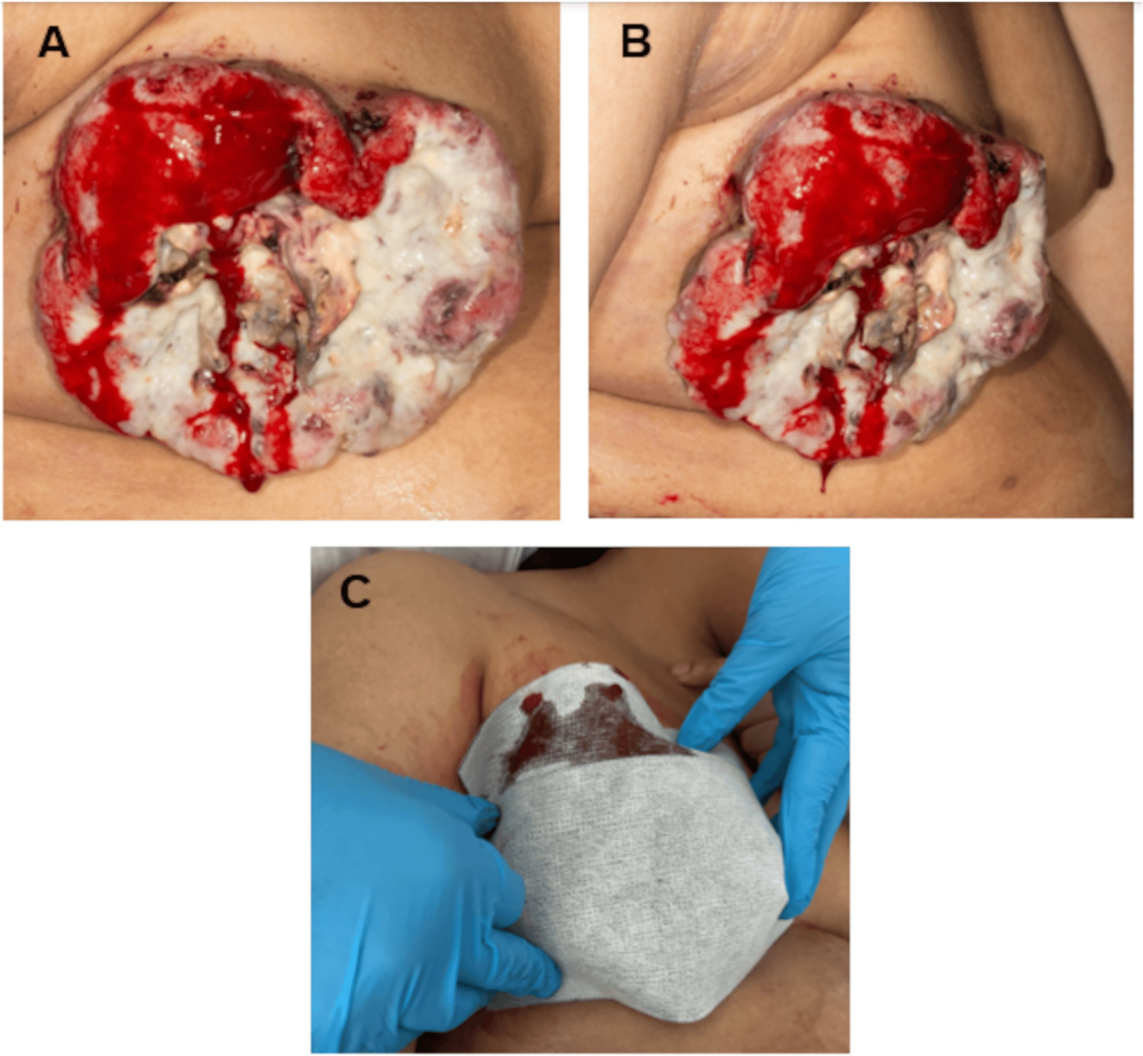
The bleeding fungating cancer on the right breast. The images show (A) anterior view, (B) lateral view, and (C) persistent bleeding from the breast despite multiple dressing changes.

The wound was initially dressed using Atrauman Ag Silver and pressure dressings; however, this failed to curtail the bleeding (Figures 1A-1C). This was followed by further unsuccessful attempts to stop the bleeding using topical adrenaline-impregnated dressing, Kaltostat Alginate hemostatic dressing, and Aquacel Extra dressings. The ongoing bleeding led to several drops in hemoglobin levels, which necessitated multiple blood transfusions over the course of the inpatient stay.

**Figure 2:**
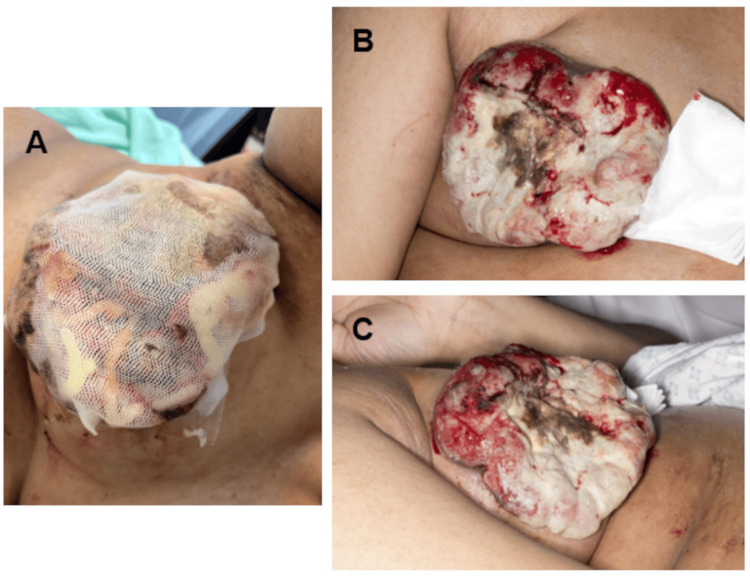
The application of Floseal Hemostatic Matrix on the bleeding fungating cancer. The images show (A) a Floseal applied, the wound covered with a breathable mesh dressing, and (B, C) dressing exposed after 24 hours, with significant improvement of bleeding compared to standard compression dressing.

Following these failed attempts, a single application of the Floseal hemostatic matrix was used and reinforced with a Zetuvit superabsorbent pad soaked in adrenaline and Kaltostat dressing (Figures 2A-2C). The immediate effect of Floseal was noted with a significant decrease in surface bleeding. One day following the application of Floseal, no further episodes of significant bleeding were encountered throughout a 16-day inpatient stay. The patient was transferred to a specialist cancer treatment center for urgent radiotherapy and systemic therapy. There have been no further reports of uncontrolled bleeding following discharge.

## Discussion

We reported a case of metastatic breast cancer presenting with profuse bleeding secondary to a malignant breast tumor. After numerous failed attempts and blood transfusions, this bleeding was controlled with a single application of a Floseal hemostatic agent. This agent is applied using an applicator or syringe that dispenses the Floseal matrix between a gauze sponge and the bleeding target; the gauze is used to hold the matrix against the bleeding surface for a period of 2 minutes [3]. Upon literature review, evidence regarding the management of bleeding in malignant wounds appears to be scarce.

Firmino et al. undertook a systematic review that sought to identify the current evidence on topical management of bleeding from breast cancer wounds [4]. They identified a total of 112 articles, of which only six were included in the review. The studies included a total of 56 patients exposed to 11 different types of topical treatments including calcium alginate, adrenaline, nonadherent dressings, silver nitrate, modified Mohs paste, 10% formalin, as well as surgical hemostasis. Table 1 summarizes the findings of the six studies included in the review [4].

**Table 1:** Summarizing the findings of the six studies included in the systemic review.

Studies	Study characteristics	Topical treatment	Outcome
Fromantin et al. [5]	Prospective study 32 patients over 42 days	Calcium alginate, epinephrine, nonadherent dressings such as 100% polyester and silicone-impregnated dressings	Bleeding was controlled at day 0 in 64% of cases, over 21 days in 84%, and over 42 days in 82%
Simman et al. [6]	Case report of 1 patient over 2 weeks	Silver nitrate, gel foam, oxidized cellulose, calcium gluconate-activated thrombin	Bleeding was controlled with calcium gluconate-activated thrombin; however, the patient eventually died from disease progression
Monleón-Just et al. [7]	Case report of 1 patient over 6 months	Metronidazole solution, silver trilaminate, hydrocellular dressing	Bleeding was improved, along with other characteristics such as exudate, odor, pain, and lesion appearance
Tsukada et al. [8]	Case report of 1 patient over 28 days	Petroleum jelly, modified Mohs paste	With 3 application cycles, the tumor shrunk in size and bleeding was controlled
Kakimoto et al. [9]	Case series of 5 patients over 3 months	Lidocaine jelly, modified Mohs paste	Active arterial bleeding was controlled within 10 minutes of application, with global bleeding, odor, and exudate improving over a timeframe of 3 weeks to 3 months
Adebamowo [10]	Case series of 16 patients over 2 years	10% formalin-soaked gauze	No significant bleeding was reported in 77.3% of the total sample, 9.1% required a second application, and 9.1% required 6-8 applications

In this review, successful hemostasis was achieved in all reported studies using different topical agents and dressing materials as shown. However, the review was limited by a small number of reported cases in noncontrolled observational studies, inconsistent methodology and patient selection across the included studies, and lack of objective assessment. Additionally, there was no strong evidence to support the choice of topical agents, preventing the review from formally endorsing or recommending any specific topical treatment.

Nicodème et al. conducted a retrospective study to assess the management of hemorrhagic malignant wounds, with breast cancer being the most frequent underlying cause [11]. Wounds were often treated with alginate or nonadherent dressings, with the former being more effective in managing spontaneous bleeding, while the latter was more effective at preventing bleeding at dressing changes. They also noted that hemorrhage episodes were fully controlled by hemostatic agents such as Pangen, Surgicel Fibrillar, and adrenaline in 70.6% of cases, with a total of 90 patients reviewed.

Simman et al. described a case in which various agents were employed to control bleeding from a malignant breast wound, including silver nitrate, gel foam, Surgicel, and thrombin activated with calcium gluconate [6]. They suggested that ligation or cauterization with silver nitrate or electrocautery may be employed where a vessel is identified; in addition, bone wax can be utilized in bleeding from the bone surface.

Though there is little mention of Floseal in the literature regarding the control of bleeding from malignant breast tumors, it has generally become one of the commonly used hemostatic agents in the surgical field. In a systematic review by Echave et al., Floseal had greater efficacy in achieving hemostasis when compared to other hemostatic agents, including Surgicel and Gelfoam [12]. Floseal has also shown promising results in obstetric surgery [13], spinal surgery [14], cardiac surgery [15], thyroid surgery [16], dental extraction [17], and epistaxis [18].

The use of hemostatic agents is not without drawbacks. False positive findings were reported at the site of topical hemostatic agents when performing PET-CT scans [19]. Henkel et al. reported the presence of microcalcifications in mammograms after the application of hemostatic products in lumpectomy cavities [20]. Nevertheless, in situations where control of life-threatening bleeding is required, this is not expected to represent a relevant concern.

## Conclusions

The present case demonstrates that Floseal, augmented with local adrenaline and appropriate dressings, can be considered for the achievement of hemostasis in cases of uncontrolled bleeding due to fungating wounds. Larger prospective or comparative studies are required to further explore these findings.
